# Epidemiology, Pathophysiology, and Management of Native Atrioventricular Valve Regurgitation in Heart Failure Patients

**DOI:** 10.3389/fcvm.2021.713658

**Published:** 2021-10-25

**Authors:** Anne-Céline Martin, Marie-Cécile Bories, Noemie Tence, Pierre Baudinaud, Louis Pechmajou, Tania Puscas, Eloi Marijon, Paul Achouh, Nicole Karam

**Affiliations:** ^1^Paris University, INSERM UMRS_1140, Paris, France; ^2^Advanced Heart Failure Unit, European Hospital Georges Pompidou, Paris, France; ^3^University of Paris, PARCC, INSERM, Paris, France; ^4^Heart Valves Unit, European Hospital Georges Pompidou, Paris, France; ^5^Electrophysiology Unit, European Hospital Georges Pompidou, Paris, France

**Keywords:** mitral regurgitation, heart failure, tricuspid regurgitation, heart–drug effects, resynchronization therapy, atrial fibrillation ablation

## Abstract

Atrioventricular regurgitation is frequent in the setting of heart failure. It is due to atrial and ventricular remodelling, as well as rhythmic disturbances and loss of synchrony. Once atrioventricular regurgitation develops, it can aggravate the underlying heart failure, and further participate and aggravate its own severity. Its presence is therefore concomitantly a surrogate of advance disease and a predictor of mortality. Heart failure management, including medical therapy, cardiac resynchronization therapy, and restoration of sinus rhythm, are the initial steps to reduce atrioventricular regurgitation. In the current review, we analyse the current data assessing the epidemiology, pathophysiology, and impact of non-valvular intervention on atrioventricular regurgitation including medical treatment, cardiac resynchronization and atrial fibrillation ablation.

## Introduction

Chronic atrioventricular valves regurgitation, whether mitral or tricuspid, is highly prevalent in the general population, and in the setting of heart failure (HF). Mitral regurgitation (MR) is currently the most common type of moderate-to-severe valve disease in the general adult population, partly due to the increase in the prevalence of treated cardiomyopathies and HF (1). Indeed, current statistics indicate that HF affects around 23 million people worldwide (2). Data from the prospective European Society of Cardiology Heart Failure Long-Term (ESC-HF-LT) Registry estimate that moderate-to-severe secondary MR is present in 36% of patients with HF with reduced ejection fraction (HFrEF), 28% with HF with mid-range ejection fraction, and 20% with HF with preserved ejection fraction (3).

The presence of secondary MR in patients with HFrEF is associated with HF symptoms, increased hospitalisation rates, and worse prognosis. Severe secondary MR is a major mortality predictor, independent of clinical and echocardiographic confounders, with an increase in mortality rate by 76 % compared to the absence of MR (4). Beyond being a surrogate of advanced cardiac disease, the presence of MR is considered as an aggravating factor (5, 6).

Similarly, tricuspid regurgitation (TR) is recognised as a common valve disease, observed in more than 1.6 million individuals in the United States. More than 80% of TR encountered in clinical practise is secondary, related to either left-sided valvular diseases, chronic atrial fibrillation, or global heart failure. In the ESC-HF-LT Registry moderate to severe TR was equally prevalent among HF subtypes, affecting approximately 20 % of the patients with HF (3). In a large cohort of patients with HFrEF, increasing TR severity was independently associated with considerably worse prognosis. Five-year survival was only 45 ± 2% for moderate TR, and 34 ± 4% for severe TR (7). Interestingly, the independent impact of TR on mortality was sustained whatever the ejection fraction was (8).

## Mitral Regurgitation

### Pathophysiology

**Figure 1:**
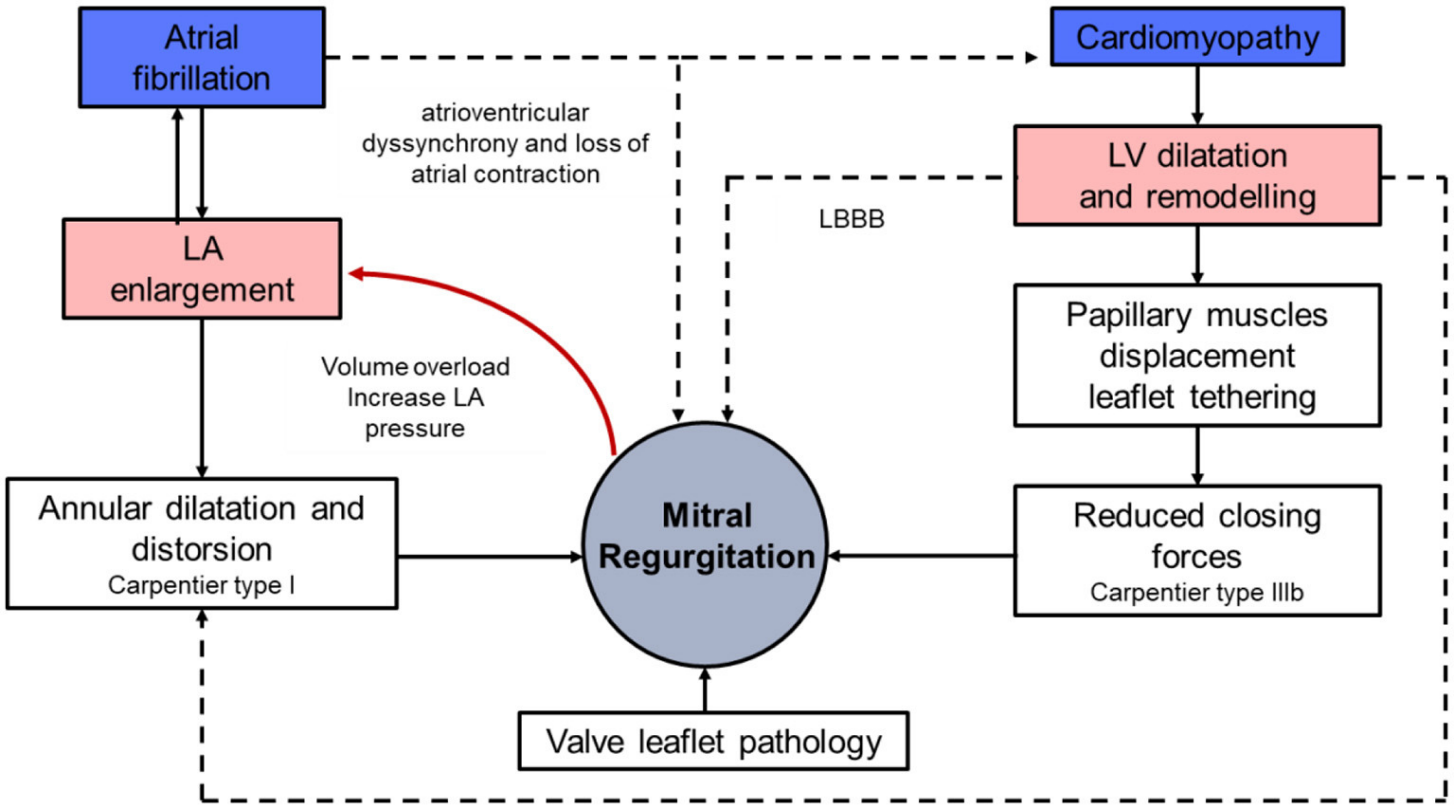
Pathophysiology of mitral regurgitation and heart failure. LA, left atrium; LV, left ventricle; LBBB, left bundle branch block.

The association of MR and HF is complex. While chronic MR can induce HF, HF can lead to progressive MR (Figure 1).

**Figure 2:**
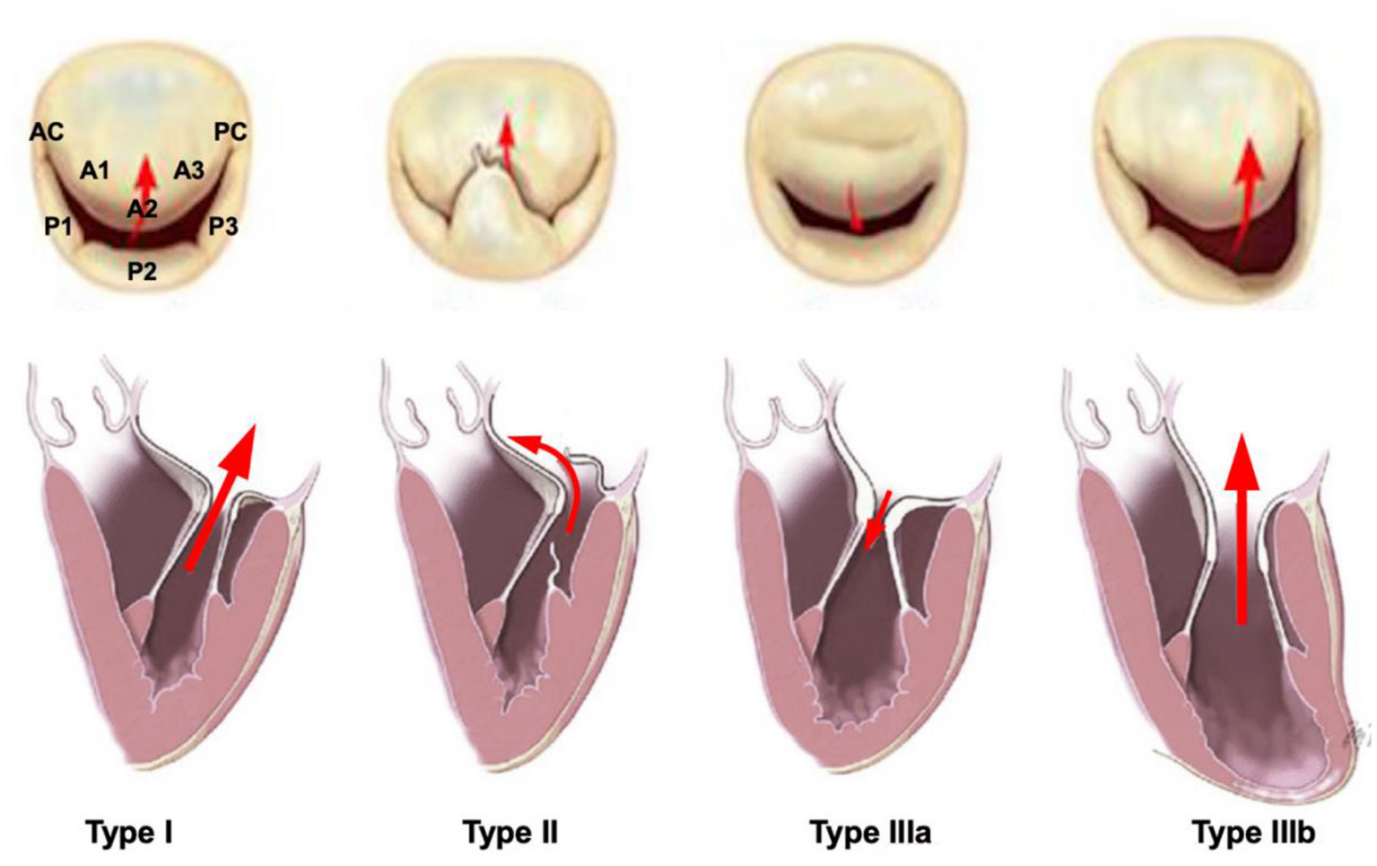
Carpentier classification for mitral valve regurgitation.

During systole, the closure of the mitral leaflets results from the interaction of closing forces (transmitral pressure gradient) and the opposing tethering forces determined by left ventricular (LV) systolic pressure. In patients with chronic HF, progressive LV remodelling induces changes in the geometry, volume and function of the LV and papillary muscle displacement, often associated with progressive annular dilation and flattening, all contributing to the development of secondary MR. Besides, focal regional LV contraction abnormalities induced by myocardial ischemia or infarction, or left bundle branch block (LBBB) can amplify the phenomenon. In the first case, desynchrony of myocardium surrounding the papillary muscles accounts for the progression of MR (9), whereas LBBB induces a delayed contraction between the segment next to anterolateral papillary muscle and the inferior segment, which results in changes in mitral tethering forces and MR, which in turn exacerbates the LV dilation. Remodelling also affects the left atrium through both the presence of MR itself, and HF-associated atrial fibrillation. Left atrial enlargement relocates the posterior mitral annulus portion and induces tethering of the posterior mitral leaflet, again increasing MR severity. Atrial fibrillation alters LV filling through the loss of atrial contraction and atrioventricular synchrony, further aggravating HF signs (10). Overall, the mechanism of MR in chronic HF combines Carpentier types I and IIIb (Figure 2). The respective part of LV damage and MR severity in HF has been presented in a new conceptual framework defined by Grayburn and al., and distinguishing, according to estimated regurgitant orifice area (EROA)/LV end diastolic volume (LVEDV) ratio, MR-dominant disease, also called “disproportionate MR,” from MR-LV-co-dominant, also referred to as “proportionate MR,” and LV-dominant, also referred to as “non-severe MR” (11).

The specificity of secondary MR in patients with HFrEF is that it is a dynamic condition, affected by changes in loading conditions, pressure or volume. Serial evaluations of MR in a given patient often reveals different degrees of severity according to the patient's status.

In the absence of underlying cardiomyopathy, patients with long-standing primary MR can develop progressive HF over time. The mechanism of HF in this case is a maladaptive remodelling, which changes the geometry, volume and function of the LV and induces papillary muscle displacement. This again will sustain and increase the severity of MR by creating a mixed-type MR, combining both the original primary MR and a secondary MR, through the mechanisms cited above.

### Therapeutic Options

Management of MR in the setting of HF depends on its underlying mechanism. In cases of primary MR leading to HF, surgical or percutaneous treatment of the mitral valve can lead to an improvement or stabilisation of HF at early stages. In the absence of early treatment, myocardial damage might become irreversible and therefore, the management will also conventional HF therapy.

#### Medical Treatment

By reversing LV remodelling, medical treatment may secondarily reduce MR severity. The cornerstone of conventional medical treatment for HFrEF involves, on top of diuretics for acute decompensations, neurohormonal therapies targeting the inhibition of the sympathetic nervous system (beta-blockers) and the renin-angiotensin-aldosterone system (RAAi), including angiotensin-converting enzyme inhibitor (ACEI), angiotensin receptor blocker (ARB), and mineralocorticoid receptor antagonist (MRA), and neprilysin inhibition with the association of sacubitril and valsartan (12). All these therapies were proven to improve HF outcome and severity. However, very few studies have specifically examined their effect on secondary MR.

Most of the data comes from beta-blockers, in particular carvedilol and metoprolol. In a double-blind randomised trial of patients with HFrEF and ischemic or dilated cardiomyopathy, metoprolol reduced LV volumes, improved LV function, and decreased mitral regurgitation in 42% of the 128 metoprolol-treated patients at 6 months, vs. 20% of the placebo-group patients (13). In 257 patients with HFrEF, carvedilol reduced the severity of MR over a 2-years follow-up in 30% of the patients. These effects were most pronounced in patients LV end-diastolic diameter >37 mm/m^2^ (14). Finally, carvedilol reduced EROA by 80% at 6 months in 45 patients with severe MR (EROA = 0.6 cm^2^) and LV diastolic diameter above 75 mm (15).

Less has been reported on the ability of RAAi to reduce MR in HFrEF. A small study (n=19 patients) showed that up-titration of lisinopril and isosorbide dinitrate in patients receiving digoxin and diuretics reduced LV end-diastolic volume leading to a MR reduction to grade 0/1 in 42% of patients with baseline severe MR at 1-year follow-up (16).

More recently, the double-blind randomised controlled trial PRIME (Pharmacological Reduction of Functional Ischemic Mitral Regurgitation) including 118 patients with HFrEF and secondary MR reported a significant reduction in both the LV end-diastolic volume and the degree of MR, as estimated by EROA and regurgitant volume, with sacubitril/valsartan, compared with valsartan at 12 months (17). Similarly, in a single centre study, switching therapy in HFrEF patients from a RAAi to sacubitril/valsartan induces beneficial reverse remodelling on LV volume and LV function at a median follow-up of 118 (77–160) days and was associated with a reduction in the degree of MR (18).

Overall, the effect of medical treatment combination on secondary MR reduction has been proven in a prospective study of 163 patients with HFrEF, whose treatment included ACEI or ARB in 85% of cases (55% receiving an optimal dose), beta-blocker in 94% (59% at optimal dose) and MRA in 43% (55% at optimal dose). Moderate or severe MR was present at baseline in 31% of the patients. Among those, 38% experienced a decrease in MR to a non-severe grade at 4 years (19). In a sub-analysis of the Cardiovascular Outcomes Assessment of the MitraClip Percutaneous Therapy for Heart Failure Patients with Functional MR (COAPT) trial comparing percutaneous mitral repair to optimal medical treatment in patients with 3+ or 4+ secondary MR, a reduction in MR severity to <2+ was seen in 34% of the 614 participants who were randomised to medical treatment (20).

Regarding other treatments recommended in selected patients with HFrEF, the SHIFT echocardiographic sub-study suggested a potential MR reduction with Ivabradine but failed in reaching significancy, despite a plausible pathophysiological rational related to LV remodelling (21). Other drugs, such as sodium-glucose cotransporter 2 (SGLT2) inhibitors and Vericiguat, a soluble guanylate cyclase stimulator, have recently joined the arsenal of HF therapy. Their benefit on LV or left atrial remodelling, and therefore on MR reduction, has not been clearly demonstrated yet.

**Figure 3:**
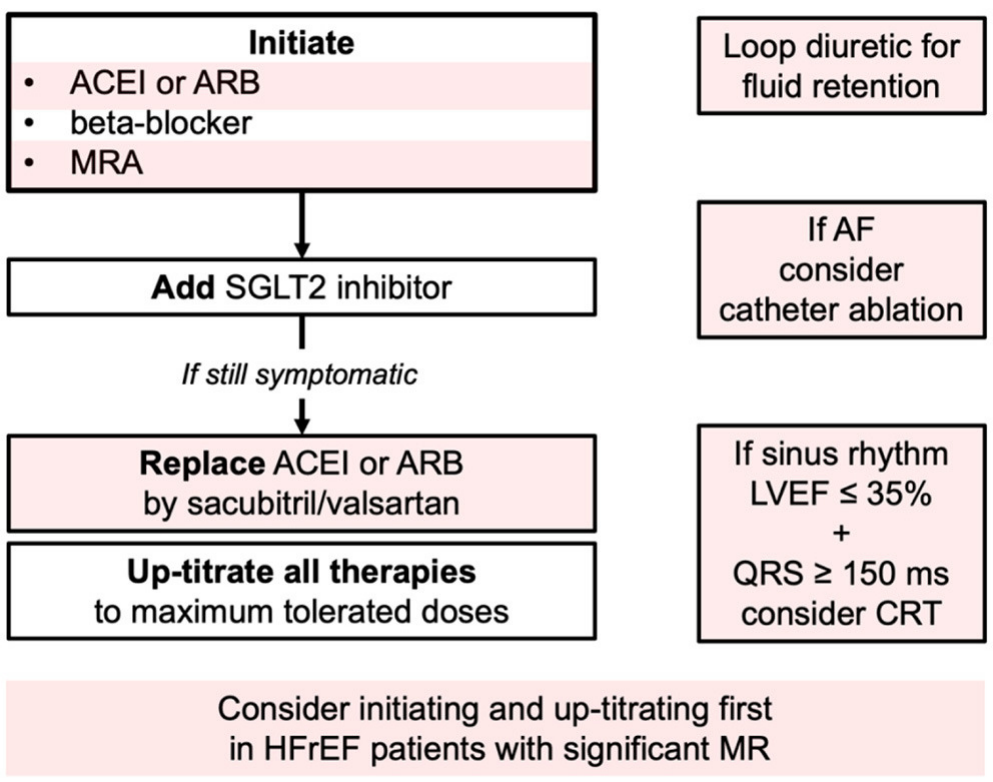
Current guideline directed approach (Class I indications 2021 ESC guidelines). HFrEF, Heart failure with reduced Ejection fraction; ACEI, angiotensin-converting enzyme inhibitor; ARB, angiotensin receptor blocker; MRA, mineralocorticoid receptor antagonist; SGLT2, sodium-glucose cotransporter 2; AF, atrial fibrillation; LVEF, left ventricle ejection fraction; CRT, cardiac resynchronization therapy; MR, mitral regurgitation.

According to the recent 2021 ESC Guidelines on the diagnosis and treatment of acute and chronic heart failure, the triad of an ACEI/sacubitril-valsartan, a beta-blocker, and an MRA is recommended as cornerstone therapies for HFrEF patients (22). These drugs should be uptitrated to the maximally tolerated doses. SGLT2 inhibitors are now recommended for all patients with HFrEF already treated with the triad regardless of whether they have diabetes or not. However, full treatment initiation is sometimes not well-tolerated, and treatments have to be introduced sequentially and progressively uptitrated. Ones suggest simultaneous initiation of treatment with a beta-blocker and an SGLT2 inhibitors, followed by addition of sacubitril/valsartan few weeks later, then MRA (Figure 3) (23). Since RAAi and sacubitril/valsartan were associated with a significant MR severity reduction, we would first suggest upgrading these molecules for patients with HFrEF and secondary MR. This proposal has to be validated in further studies.

#### Cardiac Resynchronization Therapy

Cardiac resynchronization therapy (CRT) is recommended for patients in sinus rhythm with LVEF ≤ 35%, a QRS duration ≥130 ms (Class IIa) or ≥150 ms (Class I) and LBBB morphology who remain symptomatic despite OMT in order to improve symptoms and reduce morbidity and mortality (12). Several studies assessing the yield of CRT on top of optimal medical treatment I HF patients showed a sustained reduction in MR severity with CRT (24–32) (Table 1). CRT acts through two different mechanisms. The first one is immediate, related to a more coordinated contraction of papillary muscle-bearing segments, leading to a decreased tethering. The early impact of CRT on MR decrease has been confirmed in a study by Brandt et al. that demonstrated that a 72-h cessation of long-term CRT led to a decline in LV systolic performance and an increase in secondary MR assessed by echocardiography (EROA 9.1 vs. 4.8 mm^2^, *p* < 0.0001, mitral regurgitant volume 16.0 vs. 7.8 mL, *p* < 0.0001) (33). The second mechanism of MR reduction after CRT occurs weeks to months after CRT implantation, and is due to reverse LV remodelling and restoration of synchronous ventricular contraction, thus increasing the closing forces and diminishing the mitral valve leaflet tethering, all facilitating leaflets coaptation (34, 35). The Cardiac Resynchronization-Heart Failure (CARE-HF) trial demonstrated a 37% reduction in death from any cause and cardiac hospitalisation in patients with HFrEF with CRT on top of optimal medical treatment. It also showed an MR reduction as illustrated by a reduction in EROA with a significant mean difference of −0.05 cm^2^ at 3 and −0.04 cm^2^ at 18 months (25). Of note, MR grade at 3 months was an independent predictor of survival. In the Multicenter InSync Randomised Clinical Evaluation (MIRACLE) trial including 450 patients with LVEF <35%, QRS duration >130 ms, CRT resulted in marked and sustained MR reduction. Mean area of the mitral regurgitant jet was 7.6 cm^2^ at baseline and median change reached −2.7 cm^2^ with CRT (26). In two studies on 85 and 240 patients with grade 3+/4+ MR, MR severity reduction was observed in 49% and 42% of the patients at 6 months, respectively (30, 35), while it remained stable in 37% and worsened in 21% in the second study.

**Table 1:** Studies evaluating MR reduction after CRT implantation.

**Study**	**No. of patients CRT-on/No. of patients in the study**	**% MR reduction (*p*-value) follow-up**
Cleland et al. (25)	409/813	34% (<0.05) at 3 M
Abraham et al. (26)	172/373	29% (<0.05) at 3 M
Cazeau et al. (27)	34/131	23% (<0.05) at 6 M
Cabrera-Bueno et al. (28)	34/176	18% (=0.189) at 12 M
Di Biase et al. (29)	275/794	MR improvement in 46% <0.0001
Verhaert et al. (30)	266/266	23% (<0.0001) at 6 M
Van Bommel et al. (31)	98/98	20% (<0.001) at 6 M
Sitges et al. (32)	57/151	41% (<0.01) at 12 M

Interestingly, persistent MR after CRT was strongly associated with poor outcomes (36). Indeed, van der Bijl et al. assessed MR severity evolution in 1,313 patients treated with CRT and defined 4 patterns (37). Of the 518 patients with moderate to severe MR at baseline, MR improved to no or mild MR in 209 (40%) and remained unchanged in 309 (60%) at 6 months, the latter group having the highest mortality rates, followed by those with worsened MR severity from no or mild MR at baseline to moderate to severe MR at 6 months. Baseline moderate to severe MR that remained unchanged at 6 months after CRT was independently associated with increased risk of mortality [hazard ratio 1.77 (1.41–2.22), *p* < 0.001].

Predictors of MR reduction after CRT are not known. However, patients with higher baseline MR severity are more likely to present with MR improvement after CRT. Moreover, considering the recent proportionality concept, Packer and Grayburn suggested that LBBB-associated intraventricular conduction delay characterises patients with disproportionate MR, and those patients might be better candidates for CRT, even though this concept remains to be proven (38). Since MR improvement at 3-month predicts CRT response and MR improvement at 12-month, Di Biase et al. suggested that interventional strategy should be proposed when MR persists at 3 months after CRT (29).

#### Cardioversion and Catheter Ablation of Atrial Fibrillation

Atrial fibrillation-induced left atrial and annular enlargement lead to atrial MR development or worsening, and further atrial remodelling and fibrosis that perpetuate atrial fibrillation and MR (39, 40). Therefore, restoration of sinus rhythm, through pharmacological measures, cardioversion or catheter ablation, might be needed to break the vicious circle. Dell'Era et al. demonstrated that sinus rhythm restauration by electric cardioversion in 73 patients with atrial fibrillation reduced MR severity through reverse LA remodelling and favourable effect on LV function that appears modulated by the atrium itself (41).

Several studies demonstrated that catheter ablation for atrial fibrillation reduce mortality and HF hospitalisation in HFrEF (42, 43), as well as secondary MR severity (44, 45). Importantly, in these studies, MR corresponds to “atrial MR,” which complicate 3–15% of chronic atrial fibrillation. Kawaji et al. observed in a retrospective study of 280 patients with LVEF <50% that atrial fibrillation catheter ablation improved several cardiac anomalies including LV dysfunction and left atrial dilation. MR was also significantly reduced, with only 37% of patients with moderate to severe MR at baseline having unchanged MR severity 5 years after catheter ablation (44). Similarly, Wu et al. recently showed in a small cohort including 54 patients a significant reduction in MR severity, left atrium size and LV volumes and function after restoring sinus rhythm with catheter ablation in patients with LV systolic dysfunction (45). Moderate to severe MR incidence rate decreased from 55.5% at baseline to only 11.1% after ablation (*p* = 0.007). Of note, atrial enlargement was the main mechanism of MR in these studies, since baseline characteristics showed left atrium size >40 mm in 80% of them but LVEF <40% in only 30%.

## Tricuspid Regurgitation

### Pathophysiology

**Figure 4:**
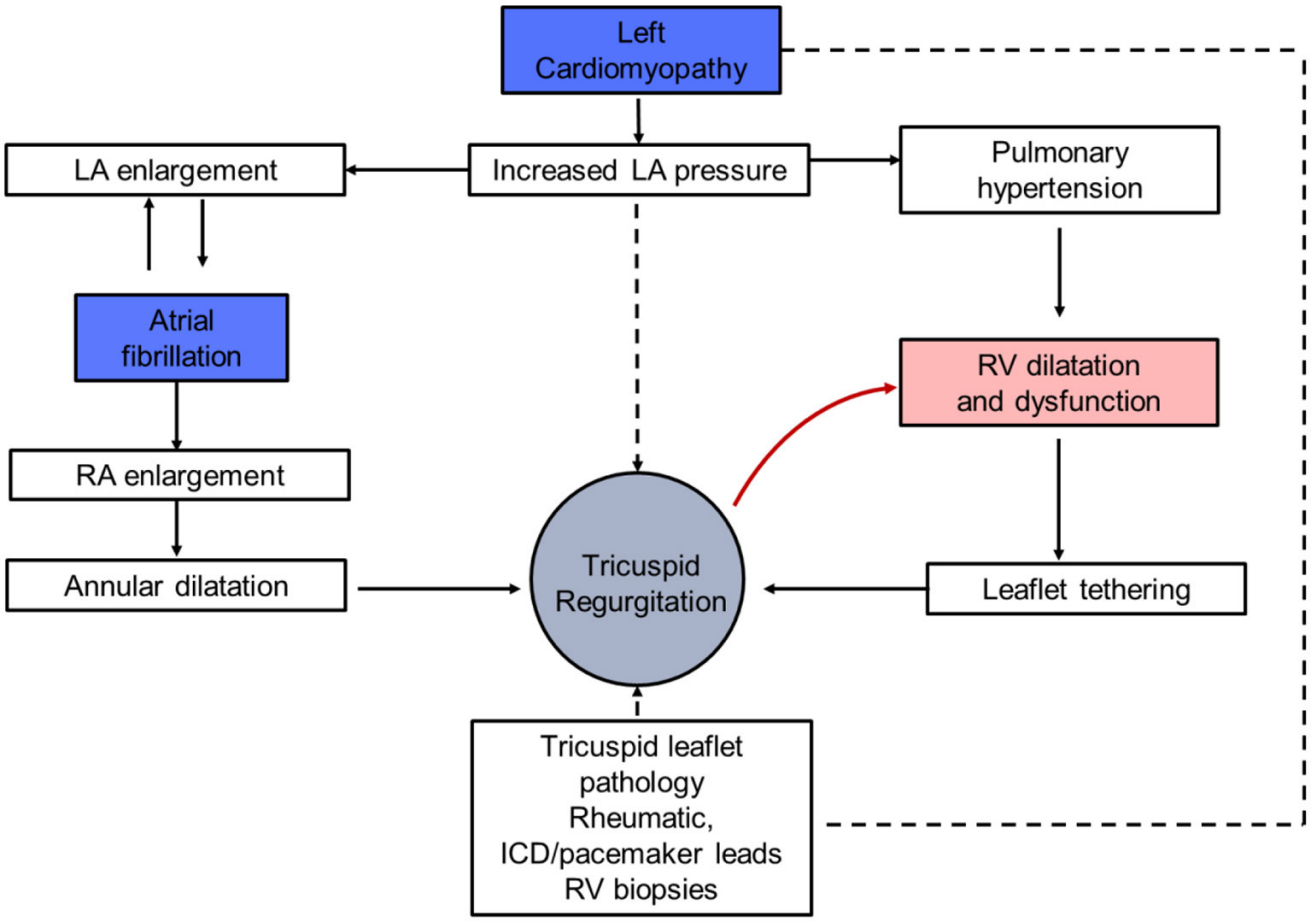
Pathophysiology of tricuspid regurgitation and heart failure. LA, left atrium; RA, right atrium; RV, right ventricle; ICD, implantable cardioverter defibrillator.

The mechanism of secondary TR in HF patients is complex and often multifactorial, frequently combining the effects of both right ventricular (RV) and right atrial dilatation (Figure 4).

The first mechanism relies on valvular leaflet tethering and restricted motion induced by RV dilatation. Indeed, left-sided HF causes increased LV filling pressures, with subsequent reduction in pulmonary artery compliance, pulmonary hypertension and overload in the RV. This leads to the dilation of right cardiac cavities and tricuspid annulus creating the substratum for TR. Since TR worsens with RV dilatation, overload promotes a vicious circle of RV failure in patients with TR and left-sided HFrEF. The second mechanism relies on tricuspid annulus dilatation as a result of the enlargement of the right atrium extending to the RV, which may result in loss of coaptation or malalignment of the leaflets then worsening of TR (46). Of note, in both situations, pacemaker and internal defibrillator leads implanted as part of HF treatment, might participate in the development or the worsening of TR (47).

### Therapeutic Options

It is mandatory to assess the characteristics of right chamber remodelling and the two types of secondary TR mechanisms for a better understanding of the pathological process leading to TR and, further, for individualising therapeutic management.

Medical treatment options for TR are very limited. Specific treatment goals include optimization of preload and afterload, and maintenance of sinus rhythm and atrioventricular synchrony.

Loop diuretics may improve symptoms while pulmonary vasodilators may lead to a reduction in TR severity. Given the dynamic behaviour of TR, like secondary MR, a transient improvement in TR severity can be observed after depletion and reduction of volume overload. In contrast, the benefit of classical HF treatments in improving TR severity is not established. ACEI has been shown to increase right ventricular ejection fraction and to reduce right ventricle end-diastolic volume and filling pressures (48). Small studies have also demonstrated that beta-blockers including carvedilol and bisoprolol improve RV systolic function (49). Some evidence supports the benefit of sinus rhythm restoration on the reduction of TR severity (50, 51). However, these studies have been performed in patients with concomitant MR and preserved ejection fraction, and whether the improvement is due to a direct impact on TR *per se* or to an improvement in MR and left heart characteristics is unknown.

## Perspectives

While the benefit of optimal medical treatment including CRT in the management of HFrEF is well-established, data show that 50% of patients respond favourably in terms of MR severity reduction (38). In case of persistent MR and unsatisfactory result of optimised medical treatment, more specific therapies should be considered.

Mitral repair with edge-to-edge percutaneous procedures can be proposed. According to the 2021 ESC/EACTS Guidelines for the management of valvular heart disease, it should be considered in selected symptomatic patients, not eligible for surgery and fulfilling criteria suggesting an increased chance of responding to the treatment (Class IIa) (22, 52). In high-risk symptomatic patients not eligible for surgery and not fulfilling the criteria suggesting an increased chance of responding to transcatheter edge-to-edge repair, the Heart Team may consider in selected cases a transcatheter edge-to-edge repair procedure or other transcatheter valve therapy if applicable, after careful evaluation for left ventricular assist device or heart transplantation. Similarly, transcatheter treatment of symptomatic secondary severe TR may be considered in inoperable patients at a Heart Valve Centre with expertise in the treatment of tricuspid valve disease. Specific treatment of TR can improve HF symptoms and right ventricular function and dimensions, as well as liver function (53–55). When TR coexists, concomitant edge-to-edge repair of the tricuspid valve in patients combining MR and TR can also be considered, knowing that it was associated with higher 1-year survival rate compared with isolated mitral repair in patients with both MR and TR (56, 57).

Left ventricle assist device (LVAD) should also be discussed early as destination therapy or as a bridge to transplantation due to its benefit on survival, functional state and quality of life (58). LVAD implantation also has drastic effects on LV remodelling leading to the reduction of LV volumes and the improvement of MR (59). It is particularly attractive in LV-dominant MR, when patients with end stage HFrEF and INTERMACS 2 to 4 status remain symptomatic despite optimal medical treatment and CRT (60) since the benefit of mitral repair seems limited. Indeed, following LVAD support, more than 80% of patients with severe MR show an improvement in MR to a level that is no longer clinically meaningful (61, 62). LVAD may also improve TR (63). Both recent 2021 Guidelines on valvular heart disease and those on heart failure agree on the necessity of considering LVAD and/or heart transplantation before considering transcatheter edge-to-edge repair in high-risk symptomatic patients with severe secondary MR and end-stage LV dysfunction (and/or RV function), not eligible for surgery and not fulfilling the criteria suggesting an increased chance of responding to transcatheter edge-to-edge repair (22, 52).

Data from the European Registry for Patients with Mechanical Circulatory Support (EUROMACS) report in 2,496 patients with LVAD support, that TR decreases immediately after implant by ~65% from moderate-to-severe TR pre-LVAD to non-to-mild TR (64). However, since moderate-to-severe TR before LVAD implantation is independently associated with poor outcomes, whether TR should be repaired before LVAD is under discussion.

## Conclusion

Non-invasive strategies including optimal medical treatment, sinus rhythm restoration in the presence of atrial fibrillation, and CRT in the case of LBBB are the first and crucial but often insufficient steps in HF patients with secondary MR or TR. The decision pathway for the management of secondary MR has been well-defined in the recent updated guidelines (22, 52). The role of heart teams composed of valve specialists, interventional cardiologists, cardiac surgeons, multimodality imaging experts, anesthesiologists and heart failure specialists is paramount in selecting the appropriate therapy for the appropriate patient. Patients have to be assessed in their entirety, taking into account the type of atrioventricular valve disease, its mechanism, the underlying myocardiopathy and the patient wishes and expectations.

## Author Contributions

NK and A-CM drafted the original manuscript. M-CB, NT, PB, LP, TP, EM, and PA reviewed the manuscript, provided comments, and suggested modifications to the manuscript. All authors contributed to the article and approved the submitted version.

## Conflict of Interest

The authors declare that the research was conducted in the absence of any commercial or financial relationships that could be construed as a potential conflict of interest.

## Publisher's Note

All claims expressed in this article are solely those of the authors and do not necessarily represent those of their affiliated organizations, or those of the publisher, the editors and the reviewers. Any product that may be evaluated in this article, or claim that may be made by its manufacturer, is not guaranteed or endorsed by the publisher.
